# Supplementary material for: A single-cell atlas of lung homeostasis reveals dynamic changes during development and aging
Source: Commun Biol. 2024 Apr 8;7:427. doi: 10.1038/s42003-024-06111-x (PMC11001898; doi:10.1038/s42003-024-06111-x)
Supplement: Supplementary file 2 — Supplementary Information [file 42003_2024_6111_MOESM2_ESM.pdf]

Supplementary material

Dynamic changes of lung homeostasis from development-to-aging single cell atlas

Hao Jia[1, *], Yuan Chang[1, *], Yulin Chen[1,2, *], Xiao Chen[1], Hang Zhang[1], Xiumeng Hua[1], Mengda Xu[1], Yixuan Sheng[1], Ningning Zhang[1], Hao Cui[1], Lei Han[1, 3], Jian Zhang[4, #], Xiaodong Fu[5, #], Jiangping Song[1, #]

[*] **First author**
[#] **Corresponding author**

1. Beijing Key Laboratory of Preclinical Research and Evaluation for Cardiovascular Implant Materials, Animal Experimental Centre, National Centre for Cardiovascular Disease, Department of Cardiac Surgery, Fuwai Hospital, Chinese Academy of Medical Sciences and Peking Union Medical College.
2. Department of Traditional Chinese and Western Medicine, Gansu University of Chinese Medicine.
3. Department of General Surgery, Yanan Hospital, Kunming Medical University.
4. Thoracic Surgery Department, the third affiliated hospital of Sun Yat-sen University, Sun Yat-sen University.
5. Department of Cardiology, Guangzhou Institute of Cardiovascular Disease, Guangdong Key Laboratory of Vascular Diseases, the Second Affiliated Hospital of Guangzhou Medical University

Jiangping Song
Beijing Key Laboratory of Preclinical Research and Evaluation for Cardiovascular Implant Materials, Animal Experimental Centre, National Centre for Cardiovascular Disease, Department of Cardiac Surgery, Fuwai Hospital, Chinese Academy of Medical Sciences and Peking Union Medical College, Beijing 100037, China. (Email: fwsongjiangping@126.com)

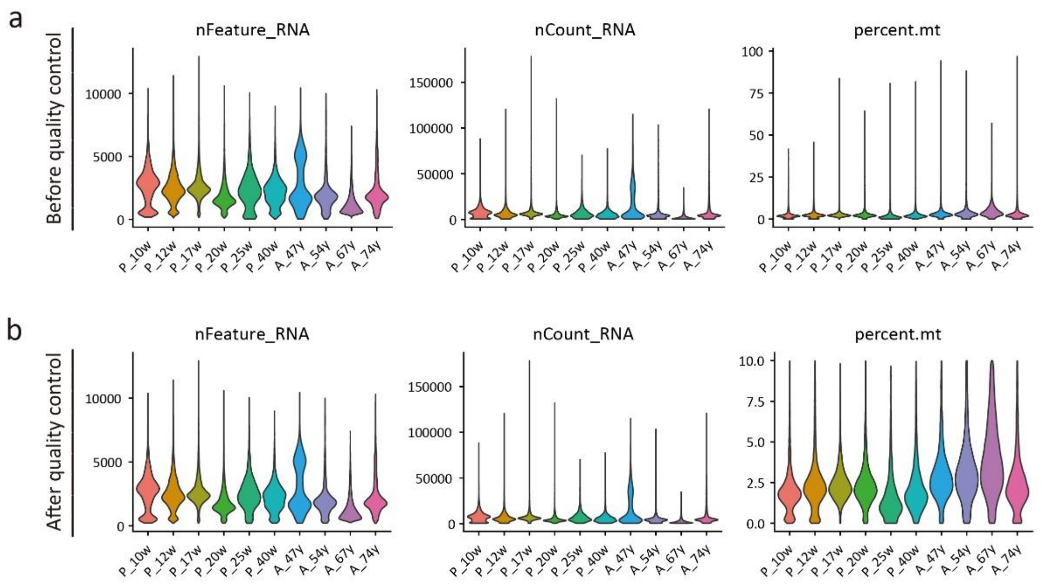

Supplementary Figure 1 | Quality control of total cell population. a. The number of genes per cell (left), number of transcripts per cell (middle), and percentage reads mapped to mitochondria per cell (right) before quality control. **b.** The number of genes per cell (left), number of transcripts per cell (middle), and percentage reads mapped to mitochondria per cell (right) after quality control.

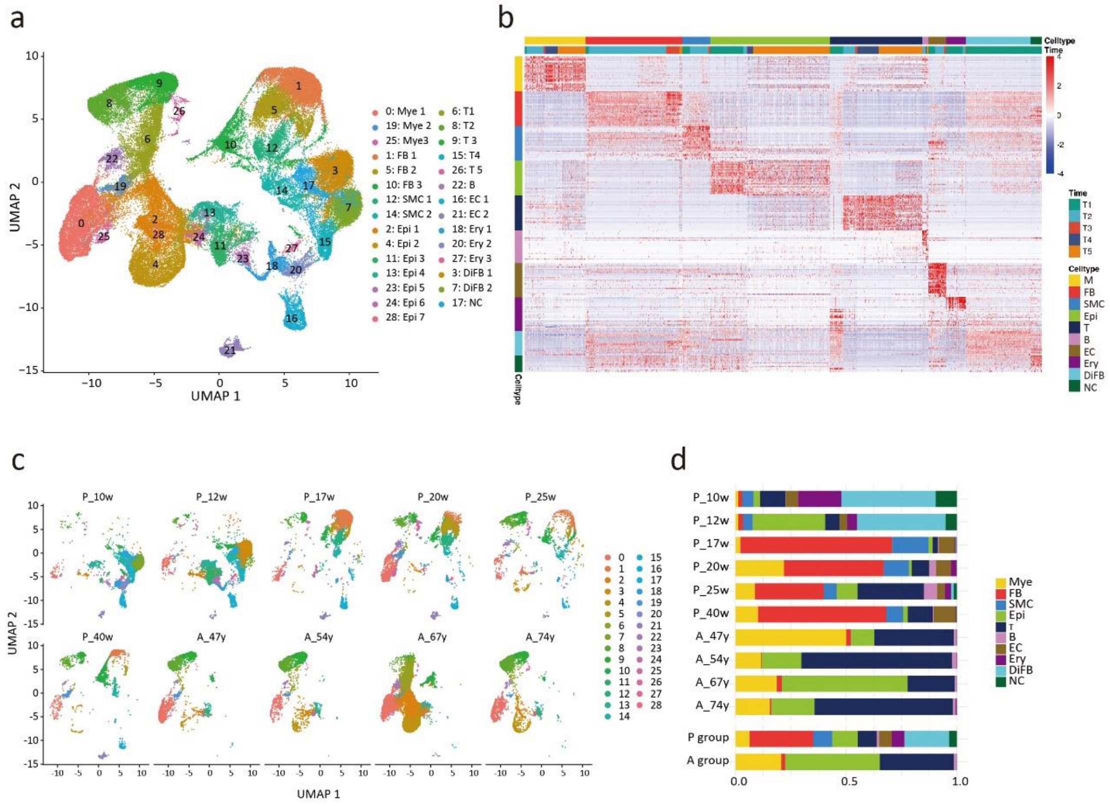

Supplementary Figure 2 | Supplementary information of the total cell population. **a.** UMAP plots showing the cell cluster distribution of the developing and aging lungs. **b.** Gene expression heatmap of the top marker genes for the 10 main cell clusters. **c.** UMAP plots showing the cell distribution in individual samples. **d.** For each of the 10 samples, the fraction of cells originating from each of the 10 main cell clusters.

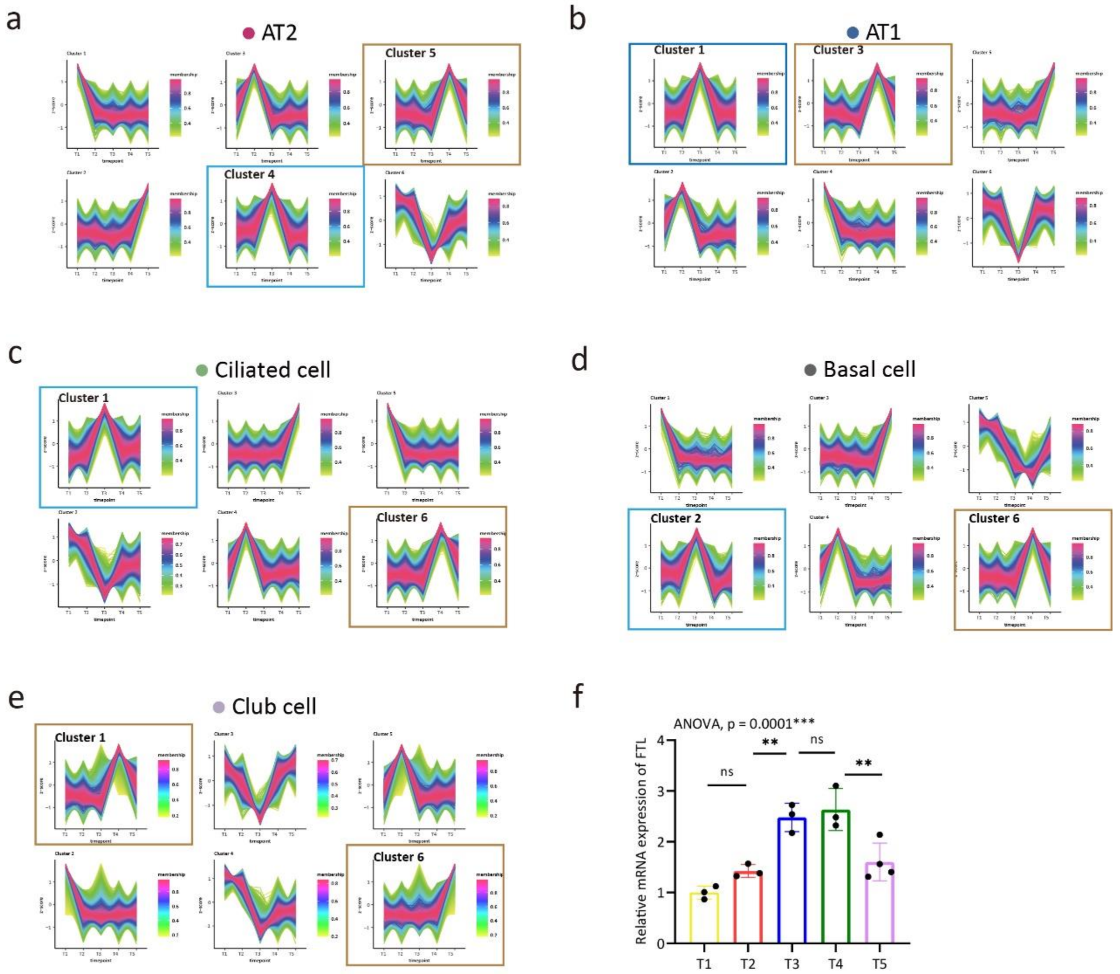

Supplementary Figure 3 | Supplementary information of epithelial cell. (a-e). Clustering of time course expression patterns of epithelial cell subclusters using the fuzzy c-means algorithm. **a.** AT2. **b**. AT1. **c.** Ciliated cell. **d.** Basal cell. **e.** Club cell. **f.** RT-qPCR analysis of relatively mRNA expression levels of FTL in 5 groups, shown as the means ± SD. The choice of ANOVA tests and two-tailed tests was based on the results of Shapiro-Wilk normality test. Dunnett tests were used to inter-group comparison. **p<0.01, ***p<0.001.

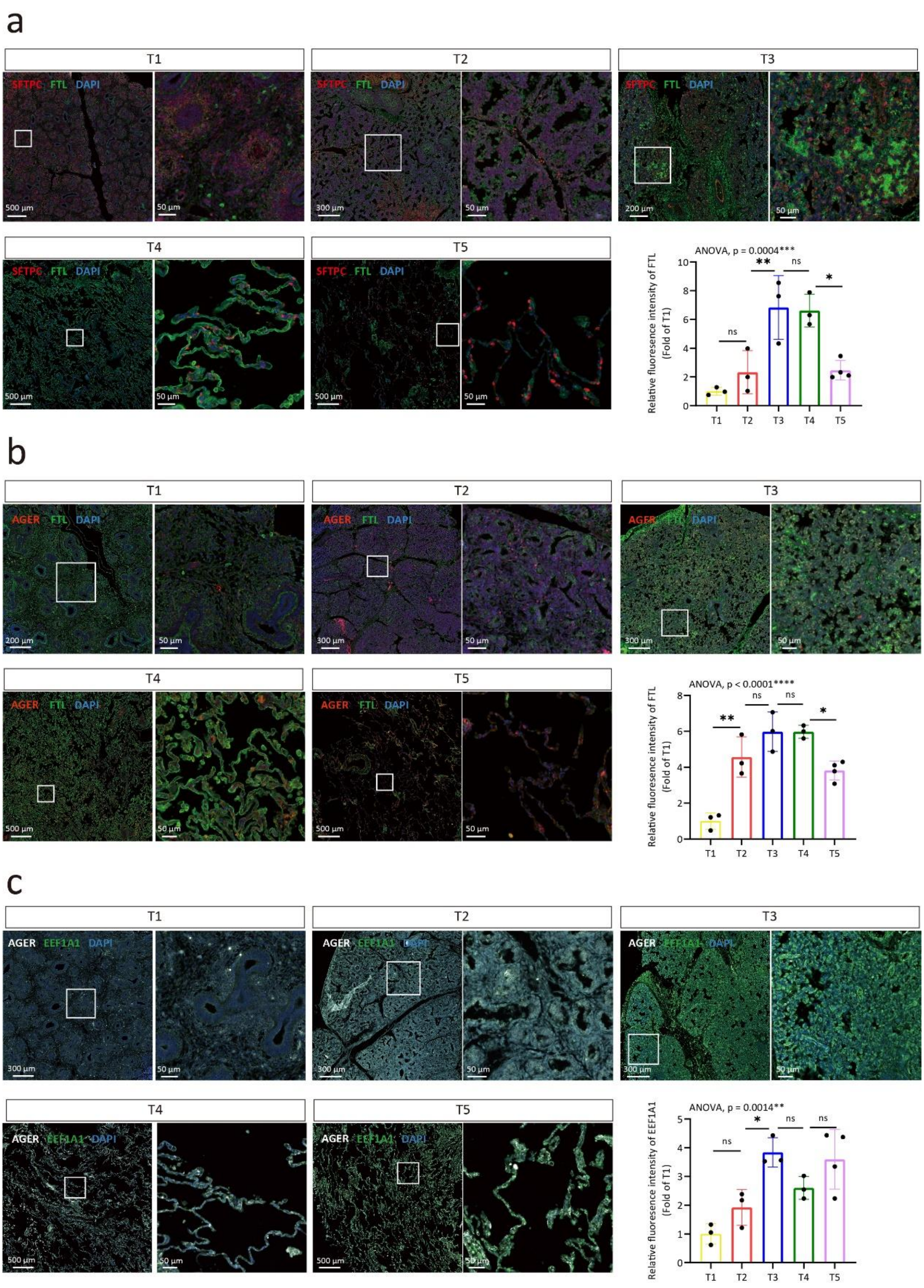

Supplementary Figure 4 | Supplementary information on the gene expression of epithelial cell. a. The expression of FTL in AT2 of immunofluorecence staining between 5 groups. **b.** The expression of FTL in AT1 of immunofluorecence staining. **c.** The expression of EEF1A1 in AT1 of immunofluorecence staining. The choice of ANOVA tests was based on the results of Shapiro-Wilk normality test. Dunnett tests were used to inter-group comparison. Data were presented as mean ± SD. *p<0.05,

**p<0.01, and ns p>0.05.

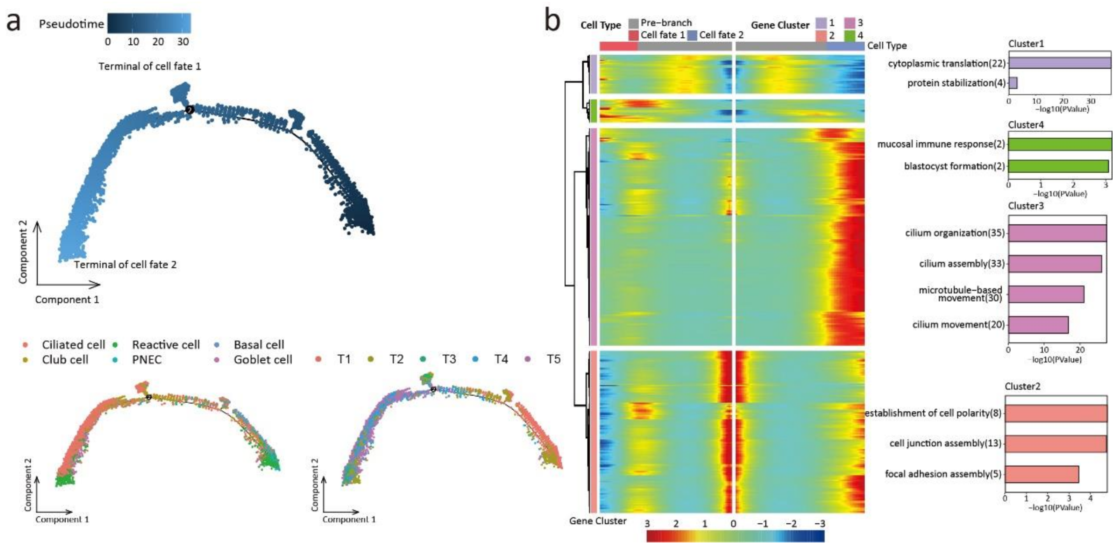

Supplementary Figure 5 | Supplementary information on the pseudotime trajectory analysis of airway epithelial cell. a. Pseudotime trajectory of airway epithelial cells inferred using monocle2 method. **b.** Heatmap showing differentially expressed genes (DEGs) alone with the pseudotime as in (a), catalogs into 4 gene module clusters (left). Gene ontology terms enriched for each gene module clusters (right).

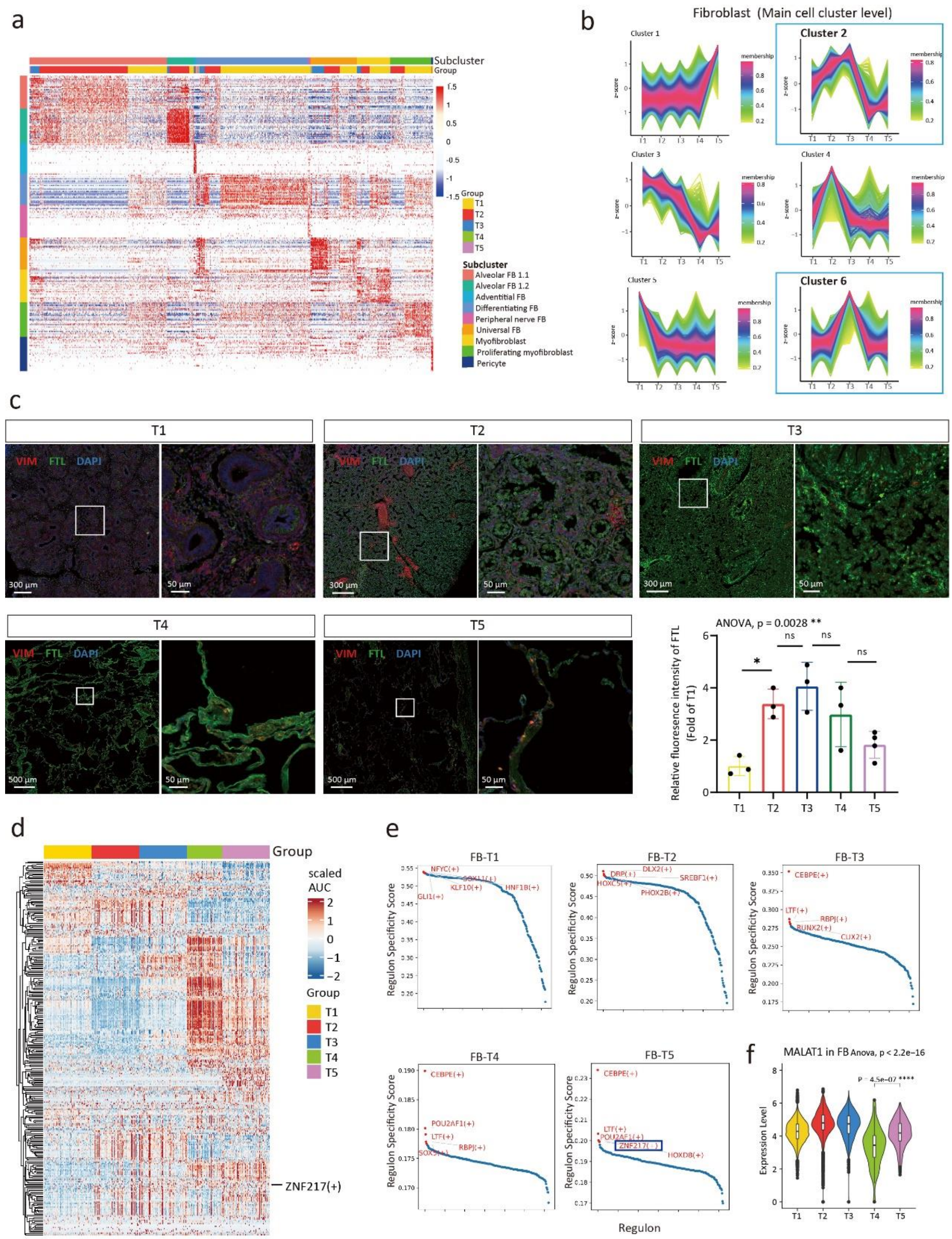

Supplementary Figure 6 | Supplementary information of fibroblast. a. Gene expression heatmap of the top marker genes for the fibroblast subclusters. **b.** Clustering of time course expression patterns of fibroblast. **c.** The expression of FTL in fibroblasts of immunofluorecence staining between 5 groups. Data were presented as mean ± SD. **d.** Heatmap of the AUC scores for the expression of genes regulated by transcription factors (TFs) in fibroblast. **e.** The regulon specificity score (RSS) of TFs in fibroblast between 5 groups. **f.** The expression level of MALAT1 in fibroblast. The choice of ANOVA tests was based on the results of Shapiro-Wilk normality test. Dunnett tests were used to inter-group comparison. *p<0.05, ****p<0.0001, and ns

$p>0.05$.

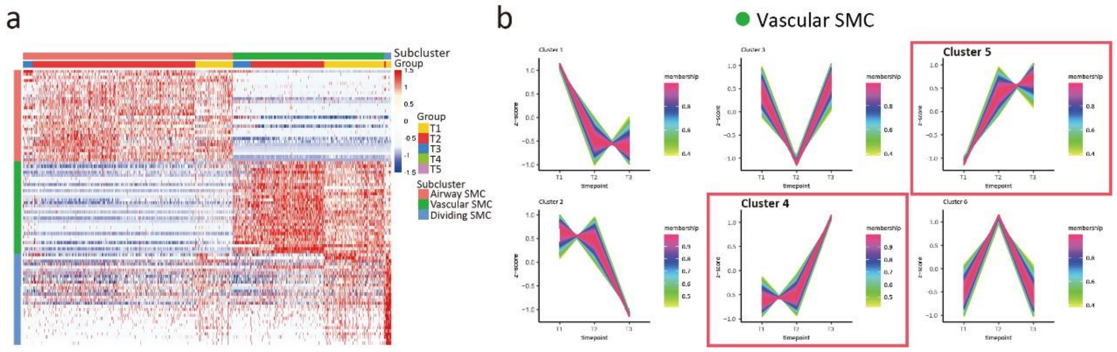

Supplementary Figure 7 | Supplementary information of smooth muscle cell. a. Gene expression heatmap of the top marker genes for the SMC subclusters. **b.** Clustering of time course expression patterns of vascular smooth muscle cell (VSMC).

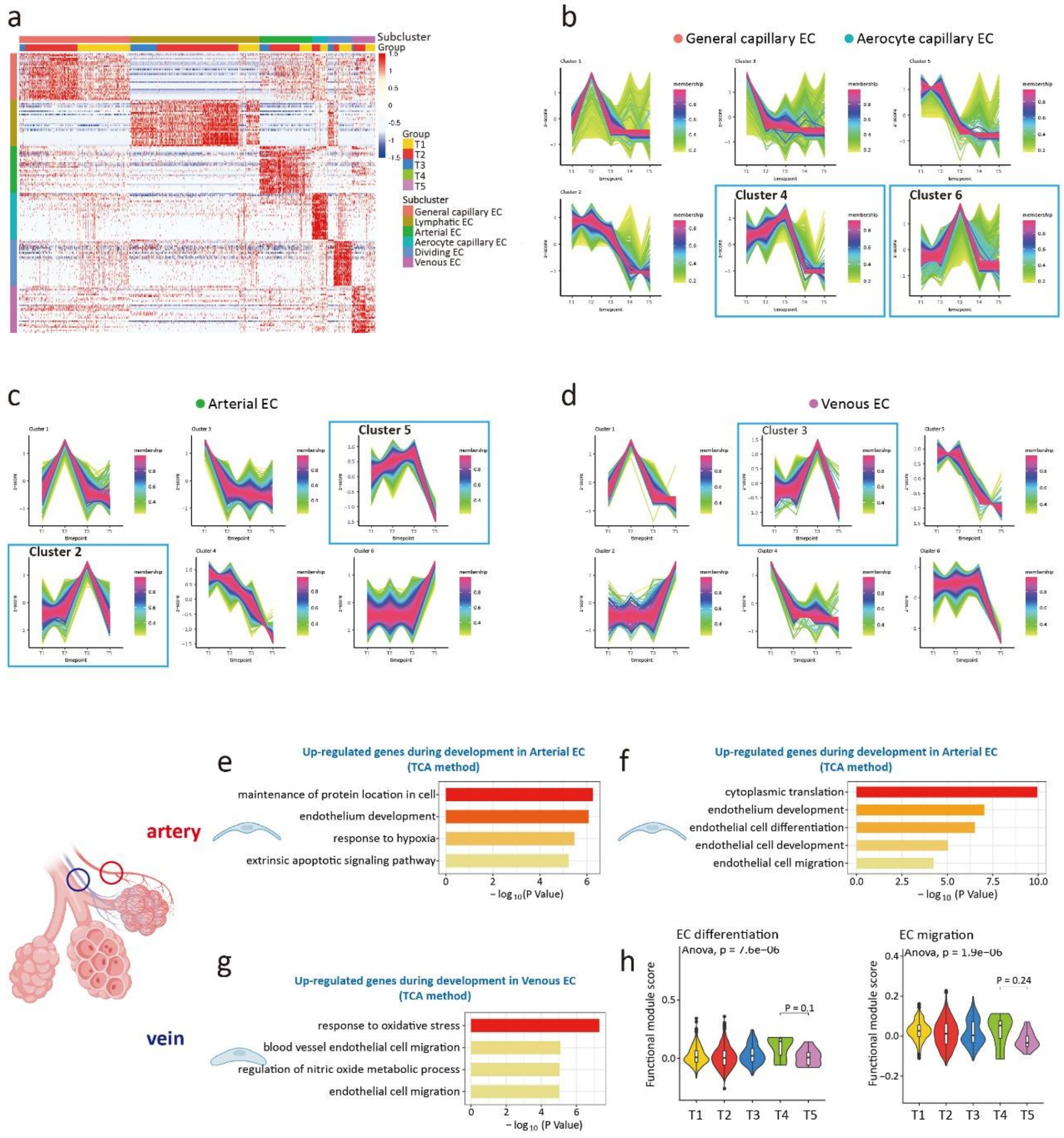

Supplementary Figure 8 | Supplementary information of endothelial cell. a. Gene expression heatmap of the top marker genes for the endothelial cell (EC) subclusters. **b.** Clustering of time course expression patterns of capillary EC. **c.** Clustering of time course expression patterns of arterial EC. **d.** Clustering of time course expression patterns of venous EC. **e.** Gene ontology enrichment terms of gene expression in cluster 2 of arterial EC (development). **f.** Gene ontology enrichment analysis of gene expression in cluster 5 of arterial EC (development). **g.** Gene ontology enrichment analysis of gene expression in cluster 3 of venous EC (development). **h.** The differentiation (GO: 0045446) scores and the migration (GO: 0043542) scores of ECs. The choice of ANOVA tests was based on the results of Shapiro-Wilk normality test. Dunnett tests were used to inter-group comparison.

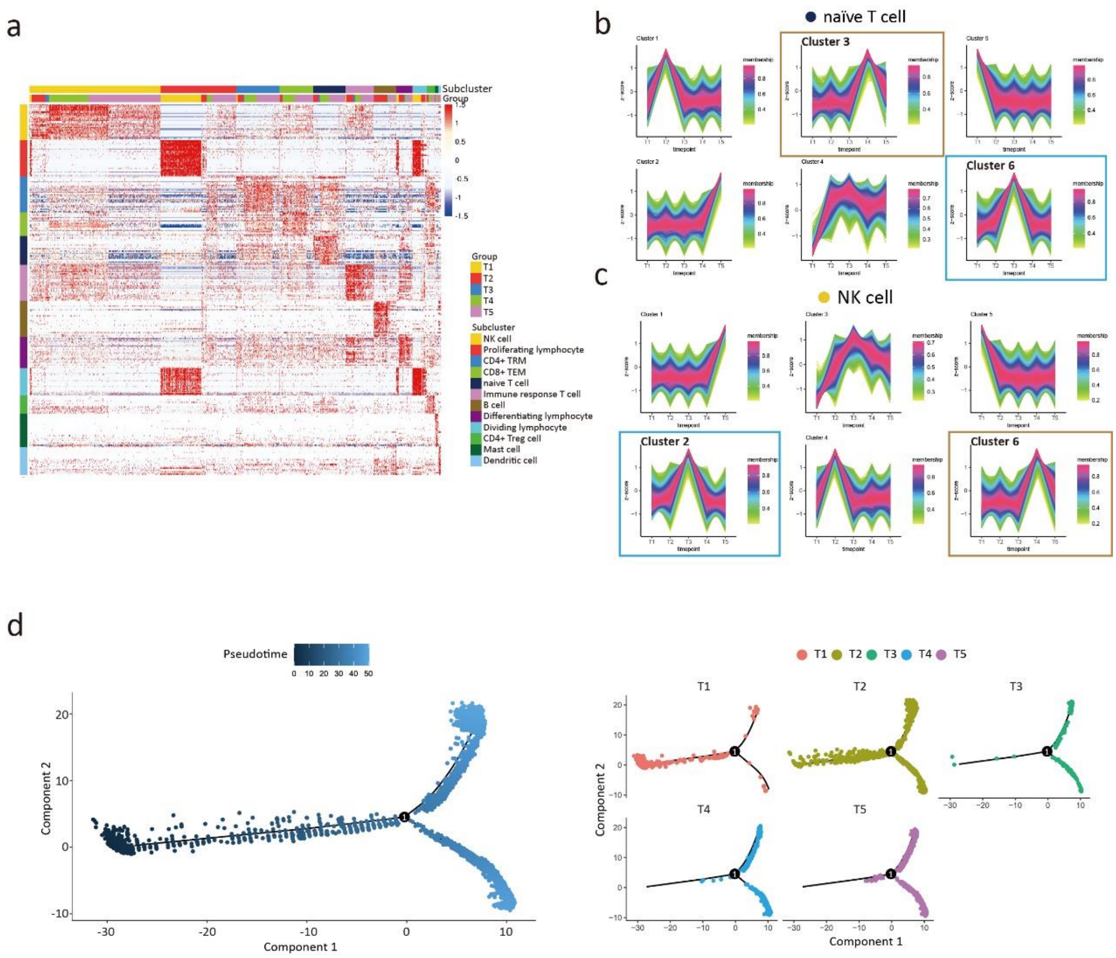

Supplementary Figure 9 | Supplementary information of lymphoid cell. a. Gene expression heatmap of the top marker genes for the lymphocyte subclusters. **b.** Clustering of time course expression patterns of naïve T cell. **c.** Clustering of time course expression patterns of NK cell subcluster. **d.** Trajectory and pseudotime of T cells inferred using monocle (left), and the phase distribution (right).

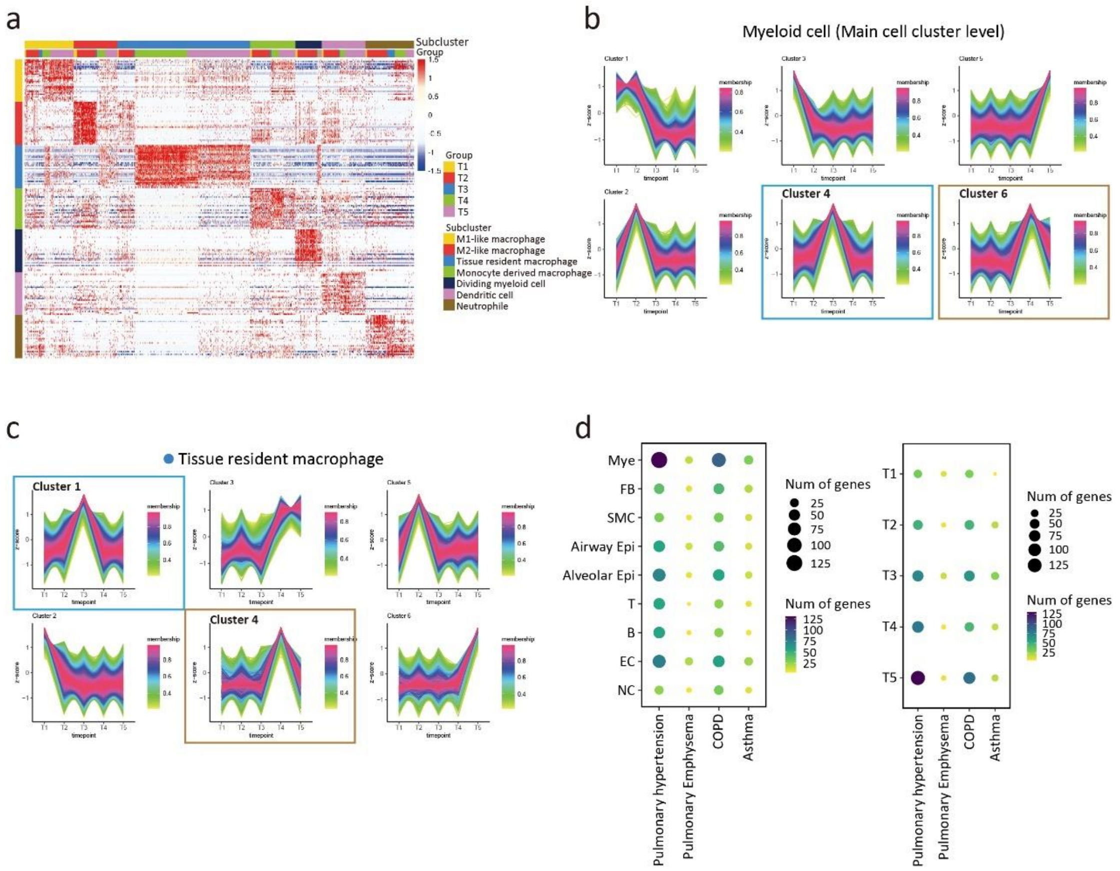

Supplementary Figure 10 | Supplementary information of myeloid cell. a. Gene expression heatmap of the top marker genes for the myeloid cell subclusters. **b.** Clustering of time course expression patterns of myeloid cell. **c.** Clustering of time course expression patterns of tissue-resident macrophage subcluster. **d.** Dot plots showing the number of genes that overlap between marker genes of main cell types and pulmonary disease-associated gene sets (left). The number of genes that overlap between marker genes of groups and pulmonary disease-associated gene sets (right).

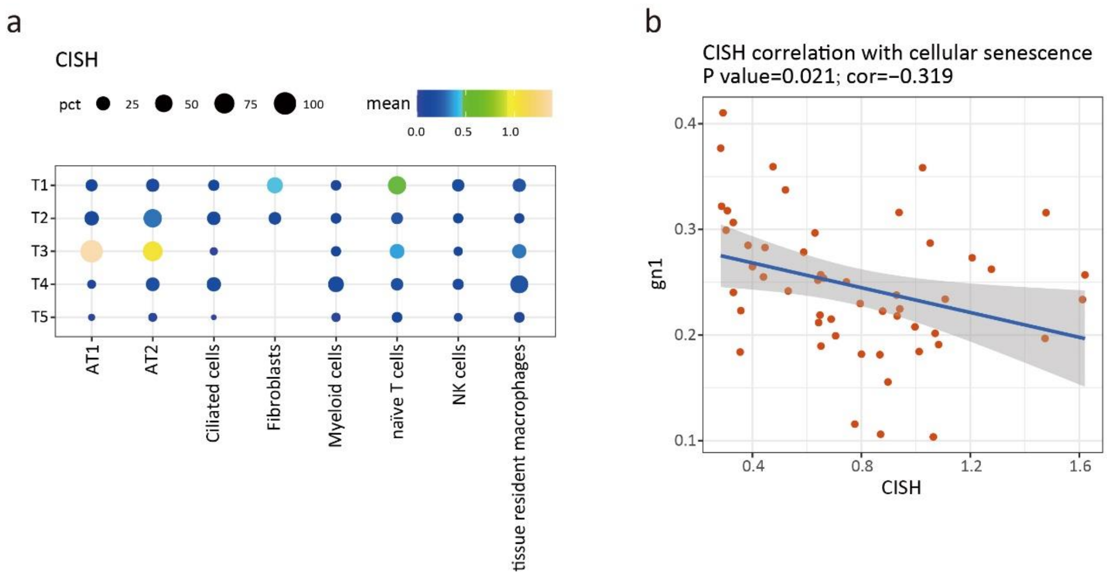

Supplementary Figure 11 | Supplementary information of CISH expression. a. Relatively expression level of CISH and the proportion of CISH-expressed cells. **b.** Correlations between the CISH expression level and the cellular senescence (GO: 0090398) score of AT2 cluster.

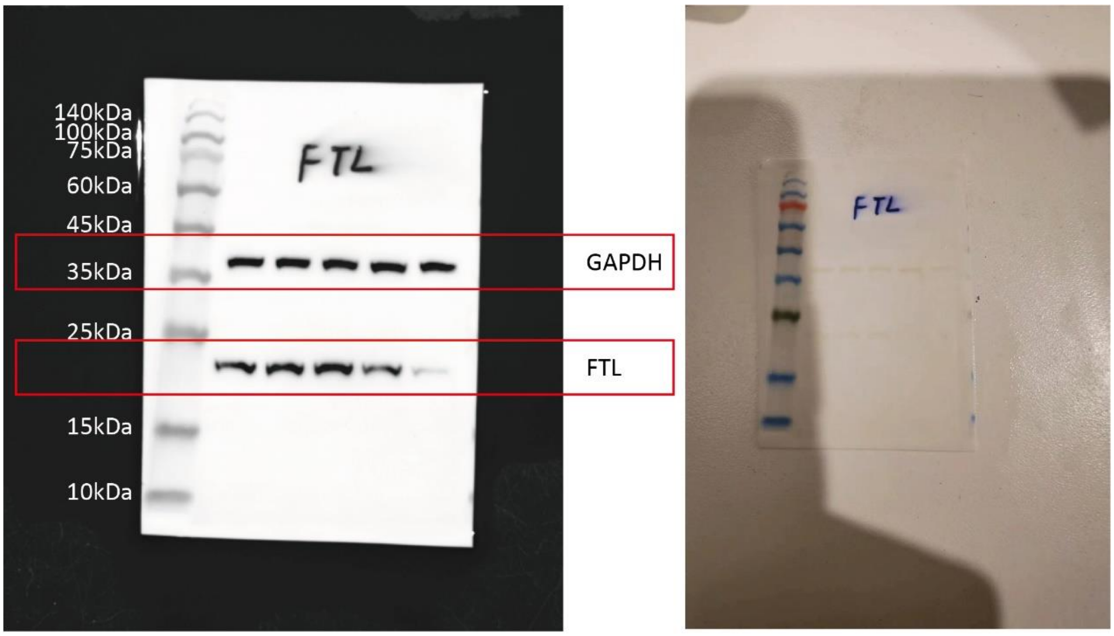

Marker (Protein Ladder): Abclonal, #RM19001, China

Supplementary Figure 12 | Supplementary information of Figure 9c. Uncropped western blot image of Figure 9c.

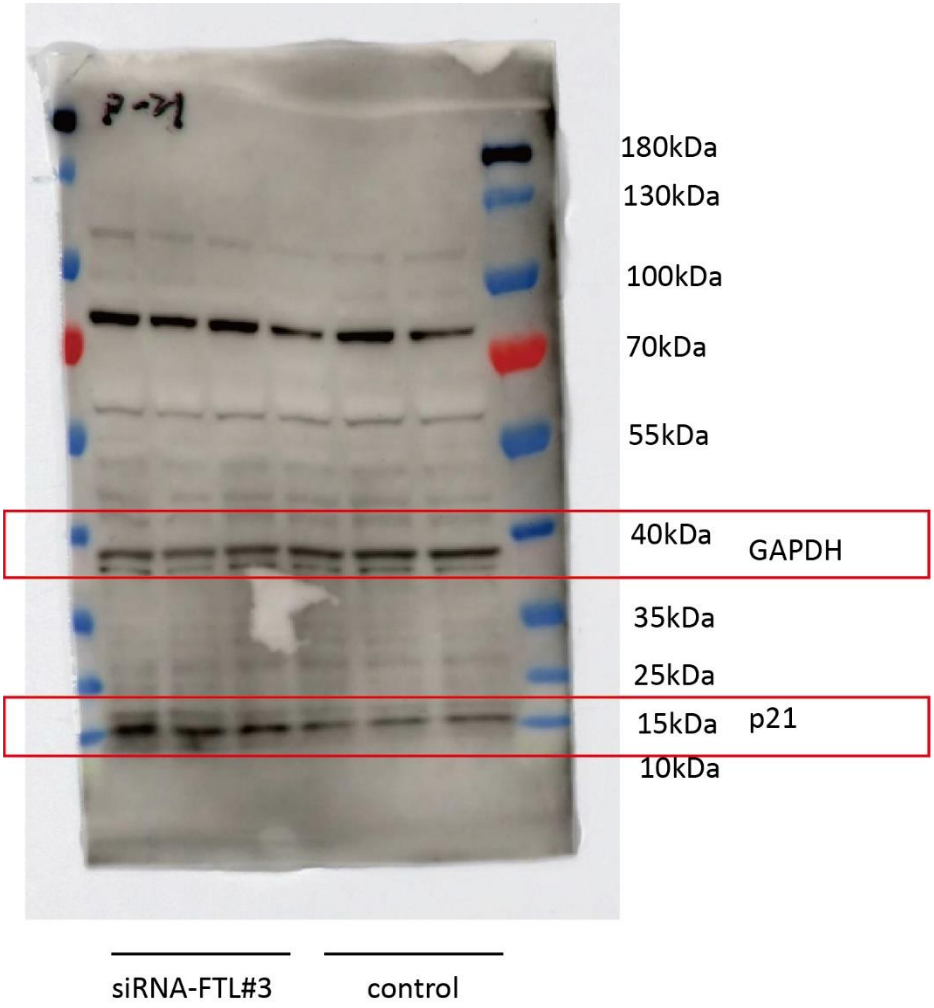

Marker (Protein Ladder): ThermoFisher Scientific, #26616, USA

Supplementary Figure 13 | Supplementary information of Figure 9f. Uncropped western blot image of Figure 9f.

Supplementary Table 1. Basic information of Samples

Sample	Gestational week	Age	Group
P_10w	10	/	T1
P_12w	12	/	T1
P_17w	17	/	T2
P_20w	20	/	T2
P_25w	25	/	T2
P_40w	40	/	T3
A_47y	/	47	T4
A_54y	/	54	T4
A_67y	/	67	T5
A_74y	/	74	T5

Supplementary Table 2. Genes used for gene scores analysis

Scores	Genes set
Elastin synthesis score	GO: 0071953 Elastic fiber
Collagen catabolic score	GO: 0030574 Collagen catabolic process
Endothelial cell proliferative score	GO: 0001935 Endothelial cell proliferation
Endothelial cell apoptotic score	GO: 0072577 Endothelial cell apoptotic process
Endothelial cell differentiation score	GO: 0045446 Endothelial cell differentiation
Endothelial cell migration score	GO: 0043542 Endothelial cell migration
Iron storage pathway score	PW: 0000592 Iron storage pathway (FTH1, FTL, FTMT, PCBP1, PCBP2)
Cellular senescence score	GO: 0090398 Cellular senescence
