# Supplementary material for: A single-cell atlas of lung homeostasis reveals dynamic changes during development and aging
Source: Commun Biol. 2024 Apr 8;7:427. doi: 10.1038/s42003-024-06111-x (PMC11001898; doi:10.1038/s42003-024-06111-x)
Supplement: Supplementary file 3 — Description of Additional Supplementary Files [file 42003_2024_6111_MOESM3_ESM.pdf]

Description of Additional Supplementary Files

File name: Supplementary Data 1

Description: Supplementary materials for unique gene sets of subclusters

File name: Supplementary Data 2

Description: Supplementary material for cell number of subclusters
